# Genome-Wide Characterization of Trehalose-6-Phosphate Synthase Gene Family of *Brassica napus* and Potential Links with Agronomic Traits

**DOI:** 10.3390/ijms232415714

**Published:** 2022-12-11

**Authors:** Ming Hu, Meili Xie, Xiaobo Cui, Junyan Huang, Xiaohui Cheng, Lijiang Liu, Shengyi Liu, Chaobo Tong

**Affiliations:** Key Laboratory of Biology and Genetics Improvement of Oil Crops, Oil Crops Research Institute of Chinese Academy of Agricultural Sciences, Ministry of Agriculture and Rural Affairs, Wuhan 430062, China

**Keywords:** trehalose-6-phosphate synthase gene family, expression pattern, association mapping analysis, agronomic traits, *Brassica napus*

## Abstract

Trehalose and trehalose-6 phosphate played important roles in floral organ development, embryonic development, cell morphogenesis, and signal transduction under abiotic stress. However, little is known about the trehalose-6-phosphate synthase (*TPS*) gene family in *Brassica napus*. In this study, in total, 26 *TPS* genes in *B. napus* (*BnTPS* genes) were identified and classified into two groups. In each group, the *BnTPS* genes showed relatively conserved gene structures. The protein–protein interaction (PPI) network and enrichment analysis indicated that *BnTPS* genes were involved in the glycolysis/gluconeogenesis, fructose and mannose metabolism, galactose metabolism, pentose phosphate pathway, carbohydrate transmembrane transport, trehalose–phosphatase activity, etc. The expression of *BnTPS* genes varied greatly across different tissues, while most of the *BnTPS* genes showed a considerable improvement in expression under different abiotic stresses, indicating that *BnTPS* genes were significantly responsive to the abiotic treatments. In addition, the association mapping analysis revealed that eight *BnTPS* genes were potential regulators of particular agronomic traits. Among them, the gene *BnTPS23* was significantly associated with the primary flowering time (PFT), full flowering time (FFT1), and final flowering time (FFT2), suggesting that *BnTPS* genes may play an important role in regulating key agronomic traits in *B. napus*. In summary, our research provides a better understanding of *BnTPS* genes, facilitates the breeding of superior *B. napus* varieties, and paves the way for future functional studies.

## 1. Introduction

Trehalose (α-D-glucopyranosyl-1, 1-α-D-glucopyranoside) is a structurally stable non-reducing disaccharide that can help biological cells maintain nucleic acid, protein, and biological membrane activity as well as protect cellular structures under adverse conditions. It consists of two glucose molecules linked by α, α, 1, and 1-glycosidic bonds [1]. Trehalose is an important signal transducer and regulator of plant metabolism and developmental processes [2,3]. It contains a large number of hydroxyl groups that can attach to a range of biomolecules and establishes hydrogen bonds, which can preserve biomolecules and lower the danger of inactivation, hence, boosting resilience to biotic or abiotic stress [1]. The trehalose-6-phosphate synthase (TPS) and trehalose-6-phosphate phosphatase (TPP) (TPS-TPP) pathway is the only one found in higher plants. The other four are the trehalose phosphorylase (TreP) pathway, trehalose synthase (TreS) pathway, maltooligosyltrehalose synthase, and maltooligosyltrehalose trehalohydrolase (MTSase–MTHase or TreY-TreZ pathway, and trehalose glycosyltransferring synthase (TreT) pathway [4,5]. TPS and TPP, two essential enzymes, work together to form the TPS-TPP pathway in a two-step reaction. TPS first catalyzes the synthesis of trehalose-6 phosphate (T6P) and UDP from UDP-glucose and glucose-6 phosphate. TPP then dephosphorylates T6P to yield trehalose [6].

TPS is crucial for the production of trehalose and stress resistance in plants [7]. In rice, TPS could improve the resistance to low temperatures, dehydration, and salt [8]. When cotton was subjected to drought, it was discovered that the expression of *TPS* genes was noticeably higher in the leaves and roots [9]. These studies showed that *TPS* genes are essential for improving plant stress tolerance and trehalose content, as well as their potential for crop improvement. Moreover, previous studies found that the role of *TPS* genes in regulating development and flowering has been reported [6]. In *A. thaliana*, the *TPS* gene regulated hypocotyl growth [10], carbohydrate utilization, and plant growth [11]. *TPS* genes can also influence the development of floral organs, such as the number of inflorescence branches and flowering time [12]. In addition, *TPS1* can regulate the flowering time by inducing the expression of FLOWERING LOCUST (FT) [13]. Furthermore, *TPS* genes can also regulate the content of sucrose and glucose in nonstructural carbohydrates (NSCs) to repress the expression of miR156 and then promote flowering [14].

Different species have different numbers of *TPS* members; for instance, the *A. thaliana* genome includes a total of 11 *TPS* genes [15], 9 in rice [16], and 7 in cucumber [17]. *B. napus*, a major global oil crop, is an allotetraploid species formed by the hybridization of *Brassica rapa* and *Brassica oleracea* [18]. However, very little is known about the *TPS* gene family in *B. napus*. In this study, the *BnTPS* gene family was identified and analyzed, including gene structure, chromosomal localization, expression, evolutionary patterns, and their potential effects on important traits. This study deepened our comprehension of *BnTPS* genes and laid the foundation for future functional studies.

## 2. Results

### 2.1. Identification and Analysis of BnTPS Genes

To identify the *BnTPS* gene family members, an HMM search by using PF00982 and PF02358 domains as queries and domain verification was performed. Finally, in total, 26 *TPS* genes were found and located in *the B. napus* genome sequence. The *BnTPS* genes were numbered according to the chromosome position and showed the putative *AtTPS* orthologs of each *BnTPS* gene based on the sequence homology (Table S1). The features of *BnTPS* genes, including isoelectric point (pI), exon number, protein length, and subcellular localization, are provided in Table S1. The length of BnTPS protein ranged from 774 to 951 amino acids (AAs), with an average length of 817 AA. The MW values ranged from 87,348.35 to 107,078.89 Da and the pI values ranged from 5.50 to 6.74. Exon number varied greatly among the *BnTPS* genes, ranging from 3 to 18. According to the subcellular location prediction, all of the BnTPS proteins were found to be located in the cytoplasm.

### 2.2. Phylogenetic Analysis and Classification of BnTPS Genes

**Figure 1:**
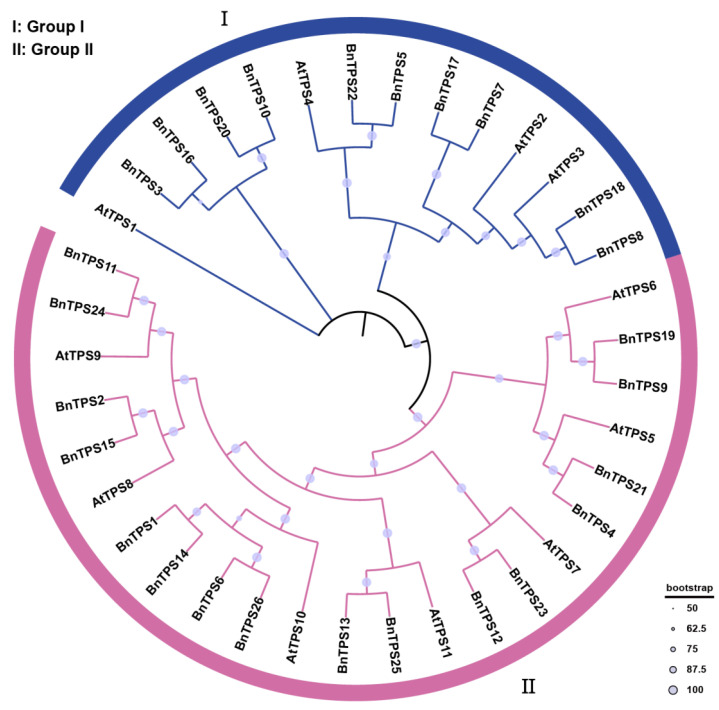
A phylogenetic tree of *BnTPS* and *AtTPS* genes. All *TPS* genes were classified into two groups and different colors represent two groups.

Using the NJ method with 1000 bootstrap replicates, a phylogenetic tree was created to assess the phylogenetic relationships among the *TPS* genes based on 26 BnTPS protein sequences and 11 *AtTPS* protein sequences (Figure 1). Based on their homologous relationship with *AtTPS* genes [15], the *TPS* genes were categorized into two groups. Most of the *BnTPS* genes were clustered in group II, where there were 16 (61.54%). The left *BnTPS* genes were clustered in group I, where there were 10 (38.46%).

### 2.3. Chromosomal Distribution and Gene Duplication of TPS Genes in Brassica napus

**Figure 2:**
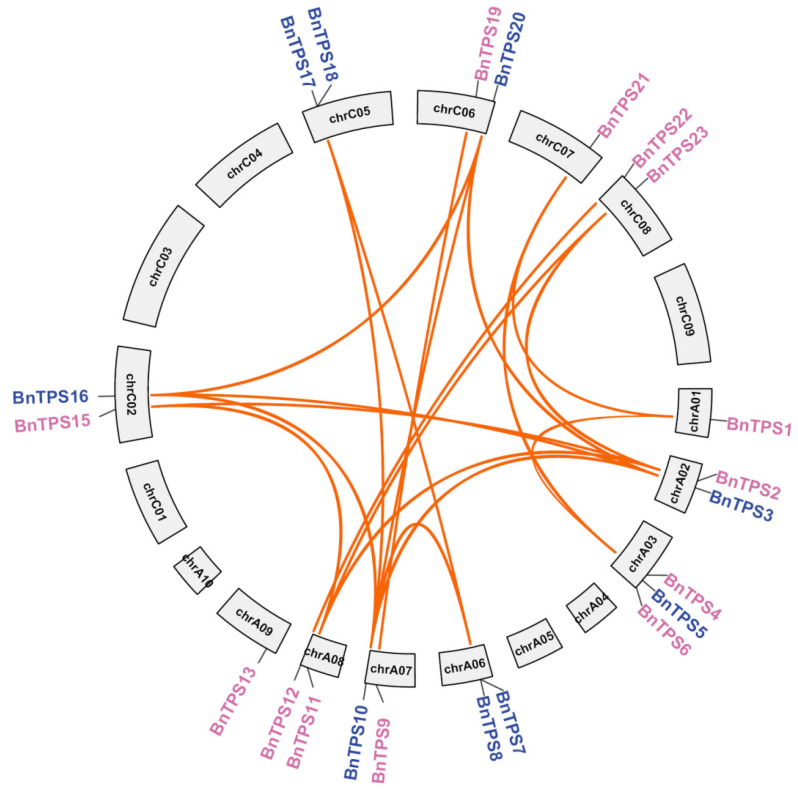
The duplicated *BnTPS* gene pairs. The genes from the two groups are indicated in pink and blue colors. The orange lines link the paralogous *BnTPS* gene pairs.

In *B. napus*, 4 out of the 26 *BnTPS* genes were not anchored in the chromosomes, while the other 22 *BnTPS* genes were unevenly distributed among 19 chromosomes (Figure 2). In total, each subgenome had 13 *BnTPS* genes, respectively (Table S1). Paralogous *BnTPS* gene pairs and the duplication patterns were identified based on the BLASTP [19] and Mcscan X software [20]. The result showed that all 26 *BnTPS* genes were derived from duplication. Most of these genes were produced from segmental duplication and whole-genome duplication (WGD) (22/26, 84.61%). Moreover, one tandem, one proximal, and two dispersed gene duplication types were also identified (Table S1). There were 20 paralogous *BnTPS* gene pairs, with 16 paralogous *BnTPS* gene pairs between the subgenome A and C, 3 in the A subgenome, and only 1 in the C subgenome (Figure 2 and Table S2). The ratios of non-synonymous to synonymous substitutions (Ka/Ks) for paralogous *BnTPS* gene pairs were analyzed to assess the selection pressure of *BnTPS* genes. In this study, all of the Ka/Ks ratios for paralogous *BnTPS* gene pairs were lower than one, showing that *BnTPS* genes underwent purifying selection (Figure 2 and Table S2).

### 2.4. Gene Structure, Conserved Motifs, and Cis-Acting Regulatory Element Analysis of BnTPS Genes

**Figure 3:**
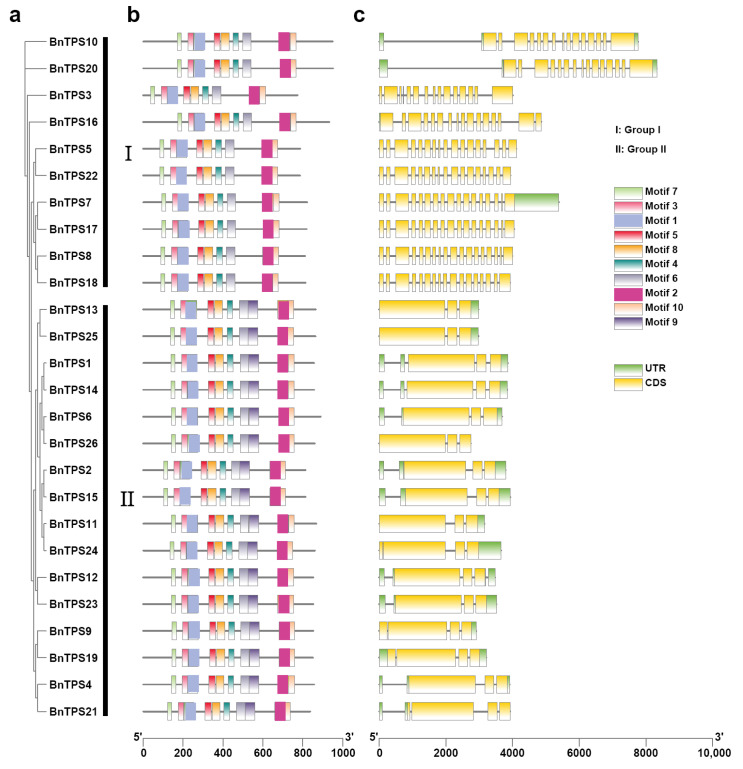
The phylogenetic tree, gene structure, and conserved motifs of 26 *BnTPS* genes. (**a**) The phylogenetic tree of 26 *BnTPS* genes; (**b**) The conversed motif composition of *BnTPS* genes. Different colors represented different motifs; (**c**) Gene structures of the *BnTPS* genes. Yellow boxes, green boxes, and grey lines represent UTR, CDS, and introns, respectively.

The exons, introns, and UTR of the *BnTPS* genes were examined to study gene structural evolution (Figure 3a,c and Table S1). Each *TPS* gene had an average of 8 exons, although the number of exons varied greatly, ranging from 3 to 18. The groups included different exons; for instance, group II only had 3 or 4 exons, while group I contained 15 to 18 exons (Figure 3c and Table S1). Furthermore, the BnTPS protein sequences were extracted from the ‘Darmor-*bzh*’ reference genome to investigate the motif composition. In total, ten conserved motifs in all of the *BnTPS* genes were found (Figure 3b). The previous study showed that the *AtTPS1* gene contained the N-terminal extension [21]. However, there were only three (*BnTPS10*, *BnTPS16*, and *BnTPS20*) of the four homologous *BnTPS* genes (*BnTPS3*, *BnTPS10*, *BnTPS16*, and *BnTPS20*) of *AtTPS1* that contained the same N-terminal extension (Figure S1 and Table S1).

**Figure 4:**
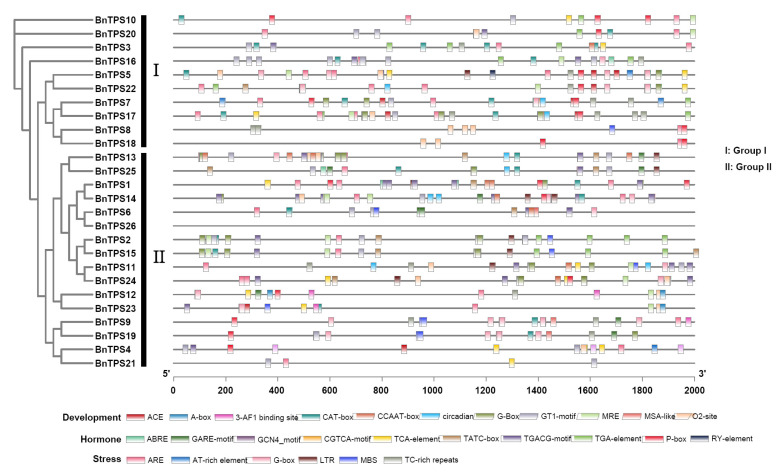
*Cis*-acting regulatory elements identified in the *BnTPS* genes promoter regions. Different colors represent various elements.

Promoter regions were proven to play an important role in regulating gene expression [22]. Therefore, we examined the *cis*-acting regulatory elements in the 2-kb promoter region of these *BnTPS* genes to ascertain the potential function. The result showed that *cis*-acting regulatory elements of *BnTPS* were found to be related to stress, development, and hormones (ranging from 4 to 44) (Figure 4 and Table S3). Most *BnTPS* genes (24/26, 92.31%) had ARE elements, important for anaerobic induction. In addition, stress-responsive elements, such as TC-rich repeats (involved in defense and stress responsiveness, 15/26, 57.69%), LTR (involved in low-temperature responsiveness, 9/26, 34.62%), and MBS (involved in drought inducibility, 8/26, 30.77%), were also common in the promoters of *BnTPS* genes. The hormone-responsive elements, such as ABRE (involved in the abscisic acid responsiveness, 17/26, 65.38%), CGTCA motif, and TGACG motif (both involved in the MeJA responsiveness, 15/26, 57.69%), existed in most of the promoters in *BnTPS* genes. In terms of development-responsive elements, CAT box (related to meristem expression, 17/26, 65.38%), GT1 motif (light responsive element, 16/26, 61.54%), and O2 site (involved in zein metabolism regulation, 12/26, 46.15%) existed in most promoters in *BnTPS* genes. These findings suggested that several *BnTPS* genes might be responsible for plant development and stress response.

### 2.5. Predicted Protein Interactions of BnTPS Proteins

**Figure 5:**
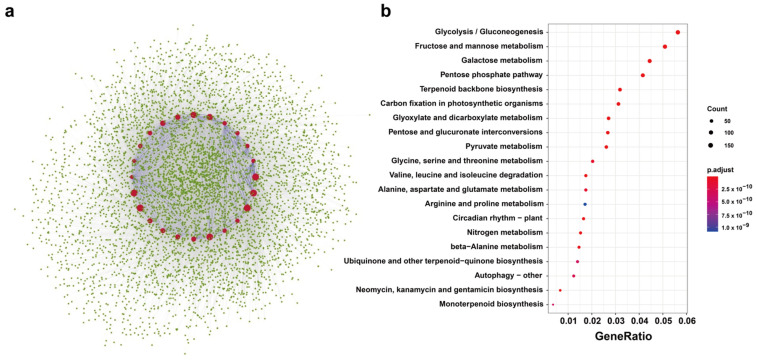
The TPS-protein interaction network analysis in *B. napus*. (**a**) The PPI network of BnTPS proteins. Red circles represent the BnTPS proteins and the green circles represent proteins that interacted with BnTPS proteins. The blue lines indicate the interaction between BnTPS proteins and the grey lines indicate the interaction between BnTPS and other proteins. (**b**) KEGG pathway analysis of proteins that interacted with BnTPS proteins. The color bar shows the p.adjust value from low (red) to high (purple).

BnTPS protein networks were modeled based on recognized protein interactions in *A. thaliana* to clarify the function of *BnTPS* genes. Using 11 AtTPS proteins as queries, 1384 proteins were found in the interactive protein database of *A. thaliana* that were homologous to 5425 proteins in *B. napus* (Figure 5a and Table S4). The result showed that most BnTPS proteins (24/26, 92.31%) were in the central nodes of the network. Most BnTPS proteins interacted with one another and with other proteins and were involved in many biological processes (Figure 5a and Table S4). Moreover, Gene Ontology (GO) and Kyoto Encyclopedia of Genes and Genomes (KEGGs) enrichment studies were performed for these interacting proteins (Figure 5b, Tables S5 and S6). The KEGG enrichment result showed the interacting genes involved in glycolysis/gluconeogenesis, fructose and mannose metabolism, galactose metabolism, pentose phosphate pathway, and so on. The GO enrichment analysis revealed that these interacted proteins were associated with various biological processes, such as carbohydrate transmembrane transport, trehalose biosynthetic process, D-ribose metabolic process, and so on (Table S5). In the cellular component terms, the interacting genes enriched in the cytosolic ribosome, 6-phosphofructokinase complex, cytoplasmic microtubule, and so on (Table S5). Meanwhile, for molecular function, the interacting genes were enriched in trehalose–phosphatase activity, polygalacturonate 4-alpha-galacturonosyltransferase activity, and so on (Table S5).

### 2.6. BnTPS Gene Expression Patterns in Different Tissues and under Different Abiotic Stresses

**Figure 6:**
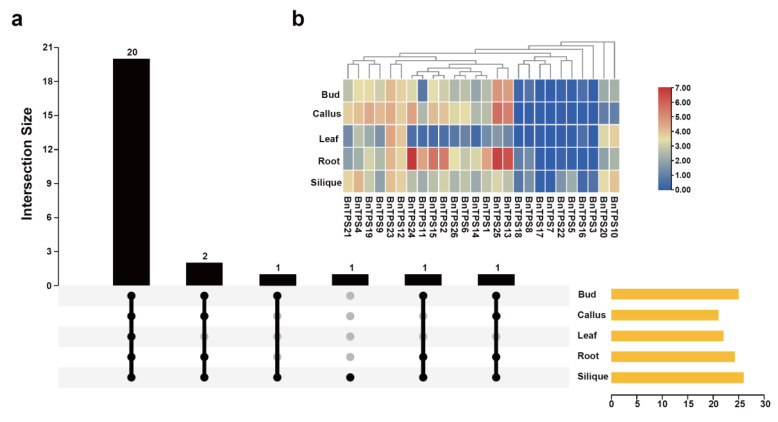
Expression profiles of *BnTPS* genes in five tissues. (**a**) The number of *BnTPS* genes that were expressed in five tissues; (**b**) Heatmap representation of 26 *BnTPS* genes in five tissues. Log2 normalization was used to process the expression data. From low (blue color) to high (red color), the color scale represents relative expression levels.

To investigate the expression pattern of the *BnTPS* gene in different tissues of *B. napus*, five tissues (bud, callus, leaf, root, and silique) of *B. napus* were used to analyze their expression pattern [23]. The result showed that *BnTPS* genes demonstrated different expression levels in these tissues. A total of 20 *BnTPS* genes was expressed in all tissues (Figure 6a and Table S7). The expression profiles of *BnTPS* genes in the bud and callus tissues showed similar patterns (Figure 6b and Table S7). Among five tissues, almost all of the *BnTPS* genes showed high expression levels in the root, bud, callus, and silique tissues but lowly expressed in leaf tissue (Figure 6b). The *BnTPS* genes showed preferential expression in the root tissue, and there were nine *BnTPS* genes that showed the highest expression levels in the root tissue (Figure 6b and Table S7). In addition, the gene *BnTPS7* was only expressed in silique tissues (Table S7).

**Figure 7:**
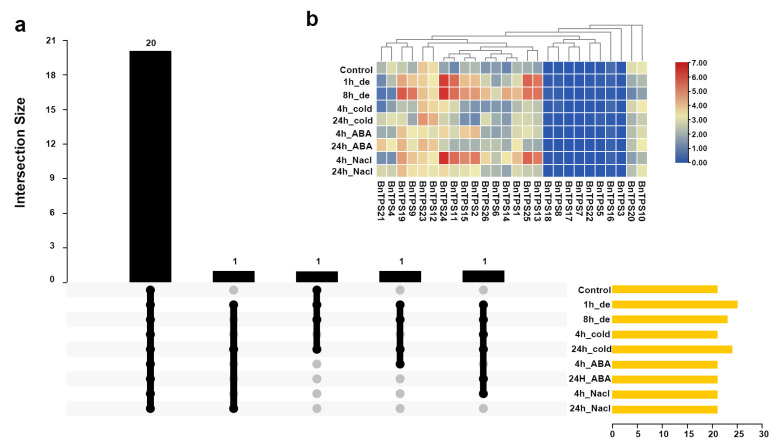
Expression profiles of *BnTPS* genes under different abiotic stress conditions. (**a**) The numbers of *BnTPS* genes that were expressed under different abiotic stress conditions; (**b**) Heatmap representation of 26 *BnTPS* genes under different abiotic stresses. Log2 normalization was used to process the expression data. From low (blue color) to high (red color), the color scale represents relative expression levels. The ‘de’ represents dehydration.

The expression pattern of *BnTPS* genes in response to abiotic stimuli, such as dehydration, cold, ABA, and salinity, was investigated. The transcriptome data were downloaded from a previous study [24]. There were 20 *BnTPS* genes expressed under all the abiotic stresses (Figure 7a and Table S8). The expressions of the majority of *BnTPS* genes were up-regulated under all the abiotic stresses (Figure 7b and Table S8). *BnTPS* gene *BnTPS11* showed increased expression (>43-fold) under dehydration and salt treatments for 4 h (Figure 7b and Table S8). Remarkably, only one *BnTPS* gene, *BnTPS20*, was down-regulated under all the abiotic stresses (Figure 7b and Table S8).

### 2.7. Genetic Effects of BnTPS Genes on Agronomic Traits

**Figure 8:**
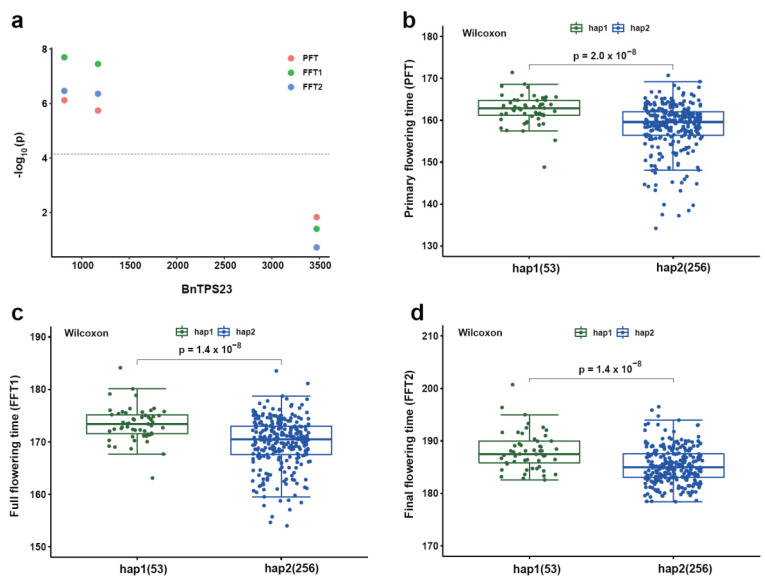
The association mapping analysis of *BnTPS23* in the *B. napus* population. (**a**) Manhattan plot of *BnTPS23* with primary flowering time (PFT), full flowering time (FFT1), and final flowering time (FFT2); (**b**–**d**) The haplotype analysis of *BnTPS23* for PFT, FFT1, and FFT2 based on the significant associated SNP.

To better understand the genetic effects of *BnTPS* genes in regulating agronomic traits, a total of 324 all-over-the-world core collections of *B. napus* germplasm were used to identify the SNPs (Table S9) [25]. In total, 655 SNPs out of 3,320,299 SNPs were found in *BnTPS* gene regions in the whole genome. All of these SNPs were annotated and showed that 434 SNPs were in the exon regions and 119 SNPs (including 99 missense mutations, 19 splicing junction mutations, and 1 stop gained mutation) resulted in producing different amino acid sequences (Table S10). On average, each *BnTPS* gene contained 25 SNPs, well above the genome-wide level (12 SNPs in each gene). The *BnTPS* genes in the A and C subgenomes were investigated separately and found that the average number of SNPs per gene within the A subgenome (41) was much higher than that of the C subgenome (10). Moreover, the average SNP number among the three groups was: group II (28) > group I (21) (Table S9). There was a significant difference between the paralogous *BnTPS* gene pairs; for example, there were 63 SNPs in *BnTPS2*, but no SNP in its paralogous gene *BnTPS24* (Table S9), suggesting that significant differences in genetic variation existed between paralogous gene pairs. To investigate the effect of *BnTPS* genes on the agronomic traits, the association mapping analysis was performed for nine agronomic traits. In total, there were 84 SNPs located in 8 *BnTPS* genes that were significantly associated with at least one important trait (Table S9). The gene (*BnTPS23*) showed a significant association with primary flowering time (PFT), full flowering time (FFT1), and final flowering time (FFT2) traits. The germplasm accessions were divided into two groups based on the significant associated SNP, and the results showed significant differences in PFT, FFT1, and FFT2 traits were observed between the two groups (Figure 8).

## 3. Discussion

It is well known that *BnTPS* genes are involved in trehalose synthesis and stress resistance in many plants [7,8,9,10]. The *TPS* gene family has been found in numerous plants [3], such as *A. thaliana* [7], rice [16], potato [26], apple [27], winter wheat [28], and cotton [29]. However, the *TPS* gene family in *B. napus* has not been extensively studied. The *Brassica* genus underwent a genome triplication after divergence from the *Arabidopsis* lineage. Subsequently, a natural cross between *B. rapa* and *B. oleracea* occurred about ~7000 years ago to form the allotetraploid *B. napus* [18]. Due to the two whole-genome duplication events, a single *A. thaliana* gene is projected to discover up to six homolog genes in *B. napus*. According to our results, *B. napus* contains only 26 *TPS* genes, while *A. thaliana* has 11 *TPS* genes (Table S1). Therefore, the number of *BnTPS* genes was only about 2.5-fold greater than that of ancestor *A. thaliana*, suggesting that the *BnTPS* genes underwent partial gene loss or sequence alteration during the evolutionary processes. For all paralogous *BnTPS* gene pairs, Ka/Ks value was less than one, suggesting that purifying selection occurred during *BnTPS* gene evolution (Table S1).

To investigate the evolution and differentiation of *BnTPS* genes, the sequence and the structure of *BnTPS* genes were compared. The *BnTPS* genes have been classified into two groups [15]. In addition, gene structure and motif analysis within the *BnTPS* genes provided additional confirmation of this classification. (Figure 3). In group I, the number of exons exceeded 15, while in group II, the number was only 3 or 4 (Figure 3 and Table S1). *BnTPS* coding sequences in the same group shared similar gene structures., suggesting that the coding sequences were conserved in group I and II.

AtTPS1 protein, which is the only Arabidopsis TPS having an N-terminal extension, contained a nuclear location signal [21,30]. There were four *BnTPS* genes (*BnTPS3*, *BnTPS10*, *BnTPS16*, and *BnTPS20*) homologous to *AtTPS1* (Table S1), indicating the common ancestry and possibly a specific function of the *BnTPS* genes. However, only three homologous *BnTPS* genes (*BnTPS10*, *BnTPS16*, and *BnTPS20*) of *AtTPS1* contained the N-terminal extension, indicating that the other one (*BnTPS3*) may have lost its N-terminal extension during evolution (Figure S1 and Table S1). *Cis*-acting elements play an important role in regulating gene expression to adapt to different environments [31]. There were some elements involved in stress (ARE, MBS, LTR), hormone (ABRE, ERE, TCA-element), and development (ACE, A-box, cat-box) found in abundance in the promoter regions of the majority of *BnTPS* genes, implying that *BnTPS* genes may play a role in stress, hormone, and development. The previous studies also showed that *TPS* genes are related to stress tolerance in *A. thaliana* and potato [3,32].

In the different tissues, the highest and the lowest average gene expression of *BnTPS* genes were observed in the root and leaf tissues separately (Figure 6 and Table S7). The root is a crucial stress-sensing tissue and *TPS* genes can regulate root development [12]; hence, *BnTPS* genes are highly expressed in root tissues. The expression divergence between the paralogous *BnTPS* gene pairs was also observed, suggesting that paralogous genes differ significantly in expression. The expression of most *BnTPS* genes varied in response to the various treatments (Figure 7 and Table S8). The expression of *BnTPS1*, *BnTPS9*, *BnTPS11*, *BnTPS13*, *BnTPS14*, *BnTPS24*, and *BnTPS25* genes increased under all the treatments (Figure 7 and Table S8). The expression of *BnTPS* genes showed a significant change under the dehydration treatment, which corresponds to previous research [33]. The expression of *BnTPS* genes in grapes did not show significant changes under cold treatment [34], but in our study, most of the *BnTPS* gene expression alterations were significant. For example, the expression of *BnTPS24* was up-regulated to 4.6-fold treated by cold treatment for 4 h, indicating that the *TPS* expression has different expression patterns in different species [34]. The *AtTPS2* gene in *A. thaliana* has an important role in regulating ABA signaling [3] and, in this study, the expression of many *BnTPS* genes changed significantly under ABA treatment for 4 h and 24 h. For example, the gene *BnTPS4* showed the largest decrease in expression under ABA treatment for 4 h, reaching 2.4-fold. In addition, under ABA treatment for 4 h, *BnTPS2* was up-regulated to 4.4-fold. Under salt treatment, *BnTPS* gene *BnTPS24* expression increased most dramatically, reaching 40.3-fold under salt treatment for 4 h, indicating the *BnTPS* genes were most sensitive to the salinity environment.

To investigate the genetic variation within the *BnTPS* gene, a worldwide core collection *B. napus* germplasm population was used to perform association mapping [24]. The result showed that SNP density in *BnTPS* genes was higher than that of the whole genome, implying that *BnTPS* genes are more dynamic. In addition, the SNP density was higher in *BnTPS* genes from the A subgenome, suggesting the asymmetric evolution of *BnTPS* genes between the A and C subgenomes. In previous studies, *TPS* genes played an important regulatory role in flower development [12,13,14,35]. The association mapping analysis was performed, and it was found that *BnTPS23* was highly significantly associated with PFT, FFT1, and FFT2. These results provided a large number of variant resources for gene function research and further breeding of elite *B. napus* varieties.

## 4. Materials and Methods

### 4.1. Identification of TPS Genes in Brassica napus

Reference genome sequence and gene annotation files were obtained from the BRAD Database (Available online: http://brassicadb.cn/, accessed on 28 September 2022) for the cultivar ‘Darmor-*bzh*’ of *B. napus* [18]. To identify the *BnTPS* genes, the TPS (Glyco-transf-20, PF00982) and TPP (Trehalose_PPase, PF02358) domains of Hidden Markov Models (HMMs) were downloaded from the Pfam database (Available online: http://pfam.xfam.org, accessed on 28 September 2022) [36]. The HMMER3.0 software was used to search for all possible *BnTPS* candidate genes, including typical TPS and TPP domains, in *B. napus* with the setting e-value of 1 × 10^−5^. Then, all candidate *BnTPS* genes were subjected to the NCBI-CDD (Conversed Domain Database) (Available online: https://www.ncbi.nlm.nih.gov/Structure/cdd/wrpsb.cgi, accessed on 28 September 2022) [37] and the SMART databases (Available online: http://smart.embl.de/, accessed on 28 September 2022) [38] to verify the presence of the TPS and TPP domains. Moreover, the remaining *BnTPS* genes were subjected to the online software ProtParam (Available online: https://web.expasy.org/protparam/, accessed on 28 September 2022) [39] to predict their molecular weights (MWs) and isoelectric point (pI), as well as instability indexes. The prediction for the subcellular location of these BnTPS proteins was conducted by CELLO v2.5 (Available online: http://cello.life.nctu.edu.tw/, accessed on 28 September 2022) [40].

### 4.2. Identification of Paralogous BnTPS Gene Pairs and Calculation of Ka/Ks Ratios

Based on the annotation of the ‘Darmor-*bzh*’ reference genome, the chromosomal locations of *TPS* genes were determined. BLASTP [19] was used to align the sequence with the setting e-value of 1 × 10^−10^, and MCScan X [20] was used to detect paralogous *BnTPS* gene pairs and the duplication patterns. Chromosomal locations and paralogous *BnTPS* gene pairs were visualized by the Circos software [41]. To determine the evolutionary pressure on paralogous genes in the *BnTPS* gene family, the ratio of non-synonymous substitution to synonymous substitution (Ka/Ks) of paralogous *BnTPS* gene pairs was calculated by Tbtools [42].

### 4.3. BnTPS Gene Structure, Conserved Motifs, and Cis-Acting Regulatory Elements Analysis

The online software MEME (Multiple Expectation Maximization for Motif Elicitation, v5.4.1) (Available online: https://meme-suite.org/meme/, accessed on 10 October 2022) was used to identify the conserved motifs in the BnTPS proteins sequence [43] with ten motif numbers. The gene structure information was shown in the genome annotation files. The Tbtools software was used to display the gene and motif structures [42]. Promoter (2 kb upstream sequences from initiation codon) sequences were extracted from genome sequence to predict the *cis*-acting regulatory elements via online software PlantCARE5 (Available online: https://bioinformatics.psb.ugent.be/webtools/plantcare/html/, accessed on 10 October 2022) [44]. The chromosomal locations of *BnTPS* genes were also displayed by Tbtools software [42].

### 4.4. Phylogenetic Analysis of TPS Genes

To reveal the evolutionary relationships of *TPS* genes, multiple sequence alignments of BnTPS and AtTPS protein sequences were performed by ClustalW software [45]. We then used the software MEGA to construct an evolutionary tree based on the result of multiple sequence alignments with the NJ method [46]. Furthermore, online software iTOL v6.5.2 (Available online: https://itol.embl.de/, accessed on 10 October 2022) [47] was used to visualize the phylogenetic tree.

### 4.5. Prediction of Protein–Protein Interactions

Based on the homologous genes in *A. thaliana*, the protein–protein interaction networks of the BnTPS proteins were predicted. The software Cytoscape [48] was used to display the PPIs that were retrieved from STRING (Available online: https://www.string-db.org/, accessed on 11 October 2022) [49]. All of these genes within the interaction underwent Gene Ontology and KEGG enrichment analysis using the R package clusterProfiler to investigate the biological functions [50].

### 4.6. Expression Pattern Analysis of BnTPS Genes

There were five tissues (leaf, callus, bud, root, and silique) and four stress conditions’ (dehydration, salt, cold, and abscisic acid) transcriptome data from previous studies [23,24]. The expression levels of *BnTPS* genes were calculated with the software Stringtie [51] after alignment with software Hisat2 [52] and then displayed by Pheatmap and UpSet in R.

### 4.7. Association Mapping of TPS Genes in a Germplasm Accession Population of B. napus

A worldwide 324 diverse *B. napus* core collection germplasm was used to investigate the potential effects of *BnTPS* genes on regulating important traits. All the SNPs in *BnTPS* gene region were annotated by the software SnpEff [53]. PFT, FFT1, FFT2, early flowering stage (EFS), late flowering stage (LFS), flowering period (FP), plant height (PH), thousand-seed weight (TSW), glucosinolate (GLU), and erucic acid were selected as studied traits [25]. The rMVP software was used for association mapping analysis between genetic variants and agronomic traits with the general linear model (GLM) method [54]. The *p* value (0.05/N) was set as the significant threshold.

## 5. Conclusions

In this study, 26 *BnTPS* genes were identified and investigated. These *BnTPS* genes were grouped into two groups. *BnTPS* genes from the same groups have similar structures and motifs. Many important *cis*-acting regulatory elements responsible for plant growth and stress response were detected in the promoters of *BnTPS* genes. The protein interaction analysis showed that *BnTPS* genes played an important role in development and stress resistance. The various expression patterns of *BnTPS* genes were revealed in different tissues and under abiotic stresses. Additionally, the association mapping results also showed that the *BnTPS* genes potentially influence agronomic traits in *B. napus*. In summary, these results offered comprehensive knowledge about *BnTPS* genes, which provided a basis for further functional research and genetic improvement for the breeding of superior *B. napus* varieties.

## Data Availability

The corresponding data are shown in Supplementary Materials.

## Supplementary Materials

The following supporting information can be downloaded at: https://www.mdpi.com/article/10.3390/ijms232415714/s1.
