# Supplementary material for: Genome-Wide Characterization of Trehalose-6-Phosphate Synthase Gene Family of Brassica napus and Potential Links with Agronomic Traits
Source: Int J Mol Sci. 2022 Dec 11;23(24):15714. doi: 10.3390/ijms232415714 (PMC9779256; doi:10.3390/ijms232415714)
Supplement: Supplementary file 1 [file ijms-23-15714-s001.zip › Supplemental Material.pdf]

```
AtTPS1     MPGNKYNCSSSHIPLSRTERLLRDRELREKRKSNRARNPNDVAGSSENSENDLRLEGDSS
BnTPS3     ------------------------------------------------------------
BnTPS16    MPGDKYNSSSSHIPLSRTERLLRERELREKRRSNRARNPNEAVGSSENSDNDLRLEGDSS
BnTPS10    MPGNKYNLSSSHIPLSRTERLLRERELREKRRSNRALNPNDNP---DNSENDLRLEGDSS
BnTPS20    MPGNKYNLSSSHIPLSRTERLLRERELRDKRRSNRALNPNDNP---DNSENDLRLEGDSS

AtTPS1     RQ-YVEQYLEGAAAAMAHDDACERQEVRPYNRQRLLVVANRLPVSAVRRGEDSWSLEISA
BnTPS3     ------------------------------------------------------------
BnTPS16    RQ-YVEQYLEGASAARAHDNVCERQEVRPYNRQRLLVVANRLPVSAVRRGEDSWSLEISA
BnTPS10    RQQYVEQYLEGAAAAMAHDDVCERQEVRPYNRQRLLVVANRLPVSAVRRGEDSWSLEISA
BnTPS20    RQQYVEQYLEGAAAAMAHDDVCERQEVRPYNRQRLLVVANRLPVSAVRRGEDSWSLEISA

AtTPS1     GGLVSALLGVKEFEARWIGWAGVNVPDEVGQKALSKALAEKRCIPVFLDEEIVHQYYNGY
BnTPS3     --------------MDWM--AGVNVPDEVGQKSLTKALAEKRCIPVFLDEEIVHQYYNGY
BnTPS16    GGLVSALLGVKEFEARWIGWAGVNVPDEVGQKALTKALAEKRCIPVFLDEEIVHQYYNGY
BnTPS10    GGLVSALLGVKEFEARWIGWAGVNVPDEVGQKALTKALAEKRCIPVFLDEEIVHQYYNGY
BnTPS20    GGLVSALLGVKEFEARWIGWAGVNVPDEVGQKALTKALAEKRCIPVFLDEEIVHQYYNGY
```

Supplemental Figure S1. Multiple sequence alignment of *TPS1* gene.
